# Rehabilitation Including Structured Active Play for Preschoolers With Cancer (RePlay)—Study Protocol for a Randomized Controlled Trial

**DOI:** 10.3389/fped.2022.834512

**Published:** 2022-05-09

**Authors:** Anna Pouplier, Helle Winther, Jan Christensen, Peter Schmidt-Andersen, He Zhang, Thomas Leth Frandsen, Kjeld Schmiegelow, Martin Kaj Fridh, Hanne Bækgaard Larsen

**Affiliations:** ^1^Department for Pediatrics and Adolescent Medicine, The Juliane Marie Center, Copenhagen University Hospital, Copenhagen, Denmark; ^2^Department of Clinical Medicine, Faculty of Health and Medical Sciences, University of Copenhagen, Copenhagen, Denmark; ^3^Department of Nutrition, Exercise and Sports, Faculty of Science, University of Copenhagen, Copenhagen, Denmark; ^4^Department of Occupational and Physiotherapy, Center of Head and Orthopedics, Copenhagen University Hospital, Copenhagen, Denmark; ^5^Mary Elizabeth's Hospital, Rigshospitalet for Children, Teens and Expecting Families, Copenhagen University Hospital, Copenhagen, Denmark

**Keywords:** preschool children, rehabilitation, structured active play, physical activity, gross motor function, social skills, randomized, pediatric oncology

## Abstract

**Background:**

Children diagnosed with cancer experience muscle weakness and impaired physical function caused by treatment and related immobility. The situation forces them into a negative cycle of diminished participation in physical and leisure activities and isolation from peers; inhibiting the natural development of social and gross motor skills. This manuscript presents a protocol for a study that explores the effects of using structured active play to maintain preschoolers' age specific gross motor function and social and personal skills while undertaking intensive cancer treatment.

**Methods:**

The study is a two-arm, superiority randomized controlled trial with an intervention and a control group designed to evaluate the effects of a structured active play intervention on gross motor function. Gross motor subtests of the Peabody Developmental Motor Scales, Second Edition (PDMS-2) are used for measurement; with the primary end-point at 6 months post-treatment initiation. Eighty-four preschool children (aged 1–5 years), newly diagnosed with cancer at the Copenhagen University Hospital are randomly assigned to either an intervention or control group, using a 1:1 allocation. The intervention group receives a combined in-hospital and home-based program that includes structured active play activities, while the control group receives standard care, including physiotherapy. During hospital admission, the intervention group undertakes 45-min structured active play group sessions three times weekly, conducted by exercise professionals. Parents receive training and supervision to facilitate daily individual sessions outside of group sessions. Secondary study outcomes target the children's overall function level in everyday life, general physical performance, and health-related quality of life. As well, children's and parents' experiences within the intervention are explored and the children's social and personal development is observed.

**Discussion:**

Limited evidence exists regarding the effectiveness of rehabilitation interventions, particularly those including active play, for preschoolers diagnosed with cancer. This manuscript reporting on a study protocol will enhance clarity and transparency in reporting and offer insights for others with interest in this same topic. Once completed, findings from this study could extend knowledge about the conduct and measurement of effectiveness in rehabilitation initiatives. If study findings suggest that the intervention is effective, structured active play may become a standard part of rehabilitation.

**Trial Registration:**

ClinicalTrials.gov: NCT04672681. Registered December 17, 2020. https://clinicaltrials.gov/ct2/show/NCT04672681.

## Introduction

Childhood cancer treatment causes multiple adverse events including myopathy, neuropathy, and loss of gross motor function (jumping, running, hopping, throwing and kicking, etc.). When experienced in parallel with treatment-related infections, inactivity and long-term bed rest this can lead to muscle weakness and decreased cardiorespiratory fitness (1–4). Such physical acute adverse events have been detected in cancer patients aged 4–18 years (1, 2, 4), and studies have shown that significantly impaired physical performance and gross motor function start in children immediately following cancer diagnosis (1, 5).

Findings suggest that younger children diagnosed with cancer (aged 6 months to 3 years) are significantly more affected by neuropathy and gross motor function impairment compared with school aged children (3). Further, interview and observational data suggest that younger children (under the age of 7) diagnosed with cancer still have a need to play, however, when visibly affected by the disease and treatment they tend to engage in sedentary play and have difficulty participating in physical activities with peers (6, 7). As such, there is an urgent need for interventions that counteract consequences of cancer treatment on gross motor function.

Previously published research indicates that physical activity and testing physical performance during cancer treatment are safe and feasible in children 6–18 years old (4, 8, 9). Moreover, physical activity positively correlates with cardiorespiratory fitness, physical function and health-related quality of life in children diagnosed with cancer (2, 4, 8, 10, 11). However, most of these studies focus on children with cancer, aged 4–18 years.

Physical activity is positively associated with bone and cardiorespiratory health, development of gross motor skills and cognition in healthy school-aged children (12–14). Developing fundamental motor function early in life ensures later development of finer motor skills (15, 16). Inadequate gross motor function in healthy preschoolers causes movement insecurity and can lead to sedentary behavior (17–20). Collectively, researchers have found significant gross motor function improvement in healthy preschoolers who followed a physical activity program compared with control groups who did not receive physical activity (16, 21–24). Yet, this has not been explored in preschoolers facing cancer treatment.

Gross motor function must be developed and reinforced through activity and interaction with others (15, 16, 20). Physical activity for preschoolers takes the form of play (25). Playing allows children to explore a world they can master, use their imagination and confronts their fears (26). Learning essential gross motor skills increases self-esteem (20) and is associated with language development, social cognition and interaction (27). Hence, gross motor function is fundamental to engaging in social and physical activities throughout the life span. Structured active play with others provides an initial arena for socialization and these peer interactions are vital to developing personal and social skills (20, 26, 28, 29). As such, structured active play is directly linked to the development of interdependent motor, social and personal skills (20, 26, 29, 30).

Taken together, cancer can impede preschoolers' fundamental gross motor function and delaying social and personal skills development. The RePlay study (Rehabilitation including structured active play for preschoolers with cancer) targets these risks in this population group. This protocol introduces the RePlay study and offer greater transparency about the study by reflecting the choices made and the expected challenges.

### Study Aims and Hypotheses

The RePlay study investigates the effectiveness of a 6-month structured active play intervention on gross motor function development in children aged 1–5 years, who are diagnosed with cancer. We hypothesize that children in the intervention group will acquire improved gross motor function compared with the children receiving standard care, in the control group.

Secondary study aims include investigation of the effectiveness of the intervention on the children's: (1) functional level in everyday life; (2) general physical performance (2 and 6 min. walk test and handgrip strength); and (3) heath-related quality of life of the children and their parents.

The study also qualitatively explores the experiences of the children and their parents. Specifically, the children's confidence in movement, joy of movement and social interactions will be probed in an effort to capture social and personal development.

We hypothesize that the intervention group will have improved gross motor function, general physical performance and health-related quality of life post-intervention compared with the standard care control group.

## Methods and Analyses

This protocol is reported according to SPIRIT (Standard Protocol Items: Recommendations for Interventional Trials) guidelines (31).

### Study Design

The study is a two-arm, superiority randomized controlled trial (RCT) with an intervention and a control group. The 6-month structured active play intervention is initiated, in parallel, with the treatment start and ends with a follow-up session 1 year post-treatment. Qualitative and quantitative data are collected and will be reported independently. The study is registered at ClinicalTrials.gov: NCT04672681.

### Setting

The study is carried out in the Department for Pediatrics and Adolescent Medicine at Copenhagen University Hospital, Denmark. All children participating in the study are screened in accordance with the study's eligibility criteria.

### Eligibility Criteria

Children eligible to participate in the study must be: (1) newly diagnosed with cancer or cancer-like benign disorders; (2) between the ages of ≥1 to <6 years at the time of diagnosis; (3) undergoing chemotherapy and/or radiation therapy at the Department for Pediatrics and Adolescent Medicine at Copenhagen University Hospital; and (4) have parents able to understand and communicate in Danish. Children considered ineligible are those diagnosed with mental disorders that prevent them from following instructions in relation to the intervention and testing.

### Recruitment

Recruitment started January 2021. Participants are recruited through referral from pediatricians in the Department for Pediatrics and Adolescent Medicine to the project nurse. Eligible participants will receive information about the study verbally, and their parents are given oral and written study information by a project nurse before providing written informed consent for their child to participate.

### Randomization

Following baseline data collection, participants are randomly placed in either the intervention or control group by means of centrally administered computer-generated (R-studio) random numbers and using a secure concealed allocation procedure. The randomization procedure is stratified by age at inclusion [respectively, <36 and ≥36 months old) and diagnostic group (hematologic malignancy, tumors located in the central nervous system (CNS tumor), and extracranial solid tumors]. The project nurse who includes the children is blinded to the randomization strategy and randomization is performed by a statistician who is not a member of the research group and who is blinded to baseline results. Due to the nature of the intervention, neither the children nor parents are blinded to their group allocation. If baseline assessment cannot be performed within two weeks of treatment initiation, randomization is carried out first, after which the children commence in either the intervention or control group without a baseline assessment.

### Structured Active Play Intervention (RePlay)

Children and their parents who are allocated to the intervention group participate in a combined in-hospital and home-based program that comprises 45 min of daily structured active play activities targeting gross motor function. In-hospital structured active play is conducted in group sessions at the hospital's pediatric physiotherapy clinic three times weekly, when the children are either admitted or visit the out-patient clinic. Outside of these group sessions, parents (or a parental figure) facilitate individual sessions for their child either in the hospital or at home. The intervention content is inspired by the Mighty Moves intervention (21). Further, the intervention was developed in collaboration with a parent panel comprising six families, each with a child aged 1–5 years and diagnosed with cancer. Different aspects of the study design were pilot tested on healthy children and children with cancer. More specifically, pilot testing of the primary outcome was done using four healthy children and two children with cancer. Additionally, two children with cancer undertook two months of the structured active play intervention prior to the inclusion process for the randomized trial. Table 1 shows the weekly schedule for the Study's intervention group.

**Table 1:** Example of a weekly schedule of structured active play for preschoolers with cancer, during treatment.

**Monday**	**Tuesday**	**Wednesday**	**Thursday**	**Friday**	**Saturday**	**Sunday**
Individual structured active play session ^*^	Individual structured active play session ^*^	Individual structured active play session ^*^	Individual structured active play session ^*^	Individual structured active play session ^*^	Individual structured active play session ^*^	Individual structured active play session ^*^
	or		or	or		
	Group structured active play session ^**^		Group structured active play session ^**^	Group structured active play session ^**^		

* *The individual sessions are conducted by the parents either at home or in the hospital room*.

** *The group sessions are conducted by an exercise professional or a trained pediatric physiotherapist*.

#### Intervention Concept

The concept of “Structured Active Play” used in this study refers specifically to instructor- or parent-led sessions of goal-oriented, age sensitive, fun movement activities that teach preschoolers gross motor skills while enhancing their social and personal skills (20, 25, 26).

#### Group Structured Active Play Sessions

Group sessions are characterized by socialization amongst children and their parents. However, the primary aim of the sessions is to enhance the children's gross motor skills as well as social and personal skills through structured active play (20, 26, 32). Each session is guided by three core principles: (1) ritual practices; (2) reinforcement of movement through repetition; and (3) development through appropriate challenge (15, 20, 33). As such, all sessions have the same start and ending ritual to establish recognizability and security for the children. Many of the activities are repeated from session to session to reinforce gross motor function development and all sessions aim to challenge the children's movement skill level. The exercise professional subjectively assesses, on a daily basis, each child's progress and sets the appropriate challenge level that can either be up- or down-scaled accordingly.

The group sessions are supervised by an exercise professional or a trained pediatric physiotherapist and take place in the pediatric occupational and physiotherapeutic clinic of the hospital. Respecting age-specific development, participants are divided into two groups: children <36 months old and those ≥36 months old. Families (including siblings if present) participate when admitted to the Department for Pediatrics and Adolescent Medicine or during appointments in the out-patient clinic. To ensure participation, the parents receive a text message reminder on the morning of each group session as well as being reminded in person. A member of the project team communicates regularly with the family and the nurse assigned to the child regarding the child's treatment and care status to ensure his/her participation in group sessions. For safety's sake, a set of daily inclusion criteria are applied to ensure that each child participating in a group session on any given day can avoid risk of an adverse event (i.e., hemoglobin >5.0 mmol/l; platelets >10 billion/l at moderate to intense activities; no active diarrhea, coughing, or cold; temperature <38.5°C; no severe comorbidities that could hinder structured active play). The treating physician and nurse are consulted prior to each child participating in a session. Admitted families treated in isolation or otherwise prevented from participating in a group session are offered an individual session with the exercise professional or pediatric physiotherapist for 45 min. These substitute sessions comprise similar activities to those in group sessions.

#### Individual Structured Active Play Sessions

On days when there are no intervention group sessions or when the children are not hospitalized, the parents facilitate individual sessions in the ward or at home. Inspirational packs are provided to assist them in facilitating active play with their child while they continue to receive oral instruction and supervision from the project team throughout the intervention. Packs include an introductory page and cards with drawings and explanations of the different activities (see introductory page and sample activities—Supplementary File 1). Many of the activities are the same as in the group sessions. Parent participation in group sessions serves to teach and inspire them to do new and old activities. Activities are color coded according to the achieved gross motor skill level of the child (34, 35). Materials are developed for two age groups: children <36 months old and those ≥36 months old; with illustrative drawings that engage the child in selecting activities that he/she prefers. Activity descriptions encourage participation by siblings and other family members.

### Control Group

Children allocated to the control group receive standard care, including physiotherapy for deficits during hospitalization. Following hospital discharge, these patients may also undergo rehabilitation, based on a deficits plan. For ethical reasons, post-intervention, those randomly allocated to the control group are offered the same inspirational material and an option to participate in the same structured active play group sessions as the intervention group families. However, they do not receive instruction or supervision for any parent-led session.

### Intervention Fidelity and Feasibility

Feasibility is assessed through acceptance, attrition, and adherence (36–38). Acceptance is defined as interest and willingness to participate in the study (i.e., the number of eligible children entering the study). Attrition is assessed by registering the number of children leaving prior to the study's completion. Adherence is measured by participation level in the intervention (i.e., monitoring of participant compliance) (36); and for which several strategies are used. Each child's participation in group sessions (including rationale for non-participation) as well as the type and duration of his/her activities are registered on standard forms. Parents are also provided a logbook for their child's individual sessions and in which they must record duration and type of activities undertaken or reasons for not doing activities.

### Outcomes

Assessments for all included children are conducted at treatment initiation (T1, baseline), 3 months after treatment initiation (T2), and at 6 months after treatment initiation (T3, post-intervention, primary end-point). Long-term follow-up will occur at 12 months after treatment initiation (T4), and 1-year post-treatment (T5). An overview of the study design, including enrollment and assessments, is summarized in Table 2.

**Table 2:** Schedule of enrollment, interventions, and assessments of participants in the RePlay study that explores gross motor function in children aged 1–5 years during intensive cancer treatment.

	**STUDY PERIOD**						
	**Enrolment**	**Pre-allocation**	**Post-allocation**				
			**Intervention**			**Follow-up**	
**Timepoints**	**TTI**	**T1 (Baseline)**	**T1^**1**^**	**T2**	**T3 (primary end-point)**	**T4**	**T5**
**ENROLMENT**							
Eligibility screen	X						
Oral and written information	X						
Informed consent		X					
**INTERVENTION**							
Structured active play			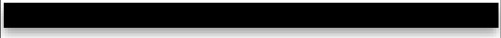				
Standard care			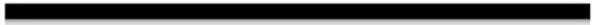				
**ASSESSMENTS**							
Medical record info		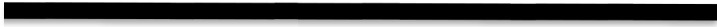					
PDMS-2		X		X	X	X	X
PEDI		X		X	X	X	X
Handgrip strength		X		X	X	X	X
6-min walk		X		X	X	X	X
Socio-demographics		X			X	X	X
PedsQL Generic Core Scale (parent proxy report)		X			X	X	X
PedsQL Cancer (parent proxy report)		X			X	X	X
PedsQL Multidimensional Fatigue Scale (parent proxy report)		X			X	X	X
RAND 36-item Health Survey		X			X	X	X
**INTERVENTION ASSESSMENTS**							
Adverse events			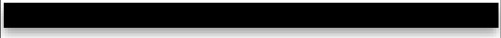				
Adherence to structured active play intervention			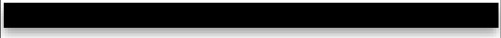				
Safety measures			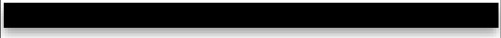				
**ADDITIONAL EMPIRICAL DATA**							
Interview					X		
Observation			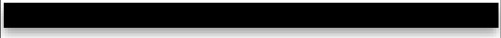				

*TTI, Treatment initiation; T1 = +1–14 days after TTI (Baseline), T1^1^= +1–14 days after TTI, T2 = 3 months (±14 days) after TTI, T3 = 6 months (±14 days) after TTI (post-intervention, primary end-point), T4 = 12 months (±14 days) after TTI, and T5 = 1 year (±14 days) after ended treatment*.

### Participant Characteristics

Upon inclusion socio-demographic information from the children and parents is collected through a questionnaire, including: place of birth, place of residence, family structure, parent admitted with the child, support from friends/relatives, daycare/preschool attendance, physical and leisure activities of the child and parents, and educational level of the parents. Furthermore, the following data are extracted from the children's medical records: sex, age, diagnosis, date of diagnosis, date of treatment initiation, date of relapse, possible date of death, comorbidity, treatment (e.g., treatment protocol, medical treatment) and medical care. In relation to daily inclusion safety criteria, data on hemoglobin level; platelets; leukocyte levels; temperature; infection symptoms and infections (organism, place, cause) are gathered from the treating physician and nurse.

### Primary Outcome Measure

#### Gross Motor Function

Gross motor function is measured using Peabody Developmental Motor Scales, Second Edition (PDMS-2), that calculates fine and gross motor function in children aged 0–6 years (39). Only the gross motor function subtests of the PDMS-2 are used in this study and consist of a total of 143 items, each scored from 0 to 2. The items are divided into three domains [i.e., stationary (30 items), locomotion (89 items) and object manipulation (24 items)] (39). The assessment is done by a trained member of the project team. The test is conducted by starting with a given item in the domain, depending on the child's age (specified in months). The items are treated singularly until the child scores 2 in three consecutive items, after which the lower level can then be determined. It may be necessary to revert to earlier items to achieve three consecutive scores of 2. The remaining items are given the score of 2. The test then proceeds with further items until the child scores 0 in three consecutive items, determining the ceiling level. The remaining items are then given the score of 0. The raw score for each domain is the sum of all the items. The test has shown acceptable psychometric properties including Cronbach α = 0.89–0.97, test-retest *r* = 0.82–0.93, and interscorer *r* = 0.96–0.99 (39, 40).

### Secondary Outcome Measures

#### Level of Everyday Function

The children's function level is determined using the Pediatric Evaluation of Disability Inventory (PEDI). The assessment is a structured interview with parents of children aged 6 months to 7 years old. PEDI is assessed by a trained member of the project team possessing comprehensive experience with the tool (41). The instrument measures capability and performance of selected functional activities on three scales: functional skills, caregiver assistance, and modifications (41, 42). In this study, the modification scale is not used. The functional skills scale is divided into three domains: self-care (73 items), mobility (59 items), and social function (65 items). Each item in the functional skill scale is scored with a 0 or 1. The caregiver assistance scale is divided into three domains: self-care (8 items), mobility (7 items) and social function (5 items). Each item in the caregiver assistance scale is scored 0 to 5 Aggregate scores are defined as the sum of each domain (42).

#### Handgrip Strength

Handgrip strength is measured using the handgrip strength test and the KLS Martin Vigorimeter (KLS Martin group, 78532 Tuttlingen, Germany) (43, 44). The instrument is a pneumatic dynamometer with three sizes of rubber bulbs. The smallest bulb is recommended for smaller children and is used for all participants in this study (43, 44). The bulb is placed with the air tube extending outward between the thumb and index finger and the fingers wrap around the bulb. The test is given twice per hand, with left and right hands alternated and a 30 s break between each trial. The children are verbally encouraged to squeeze the bulb as hard as they can. All scores (kPa) are registered and the highest score for each hand is used for the analysis.

#### Six-Minute Walk Test

The six-minute walk test (6MWT) is a general physical performance indicator (45). The test is done in a hallway with a 15-meter track that is clearly marked at each end. The child is asked to walk from one mark to the other as many times as he/she can within 6 min. The assessor verbally encourages the child to keep walking during the 6 min; if needed, parents can walk beside their child along the track and are reminded that it is the child who sets the pace. Given that the 6MWT with younger children whose concentration is limited can be difficult there will also be a recording of the distance walked after the initial 2 minutes (2MWT) in case they can't walk all 6 minutes. The 2MWT is validated in younger children (46, 47).

#### Health-Related Quality of Life (HRQoL)

HRQoL of the children and parents are measured through four standardized questionnaires. HRQoL of the children is measured through three parent proxy-report questionnaires designed to assess the parent's perceptions of their child's health-related quality of life. The PedsQL Generic Core Scale measures quality of life using 21 items in 4 domains; physical, emotional and social function; and school/preschool activity (48). PedsQL Cancer measures the effect of cancer on the child using 25 items in 8 domains: pain, nausea, procedure anxiety, treatment anxiety, worry, cognitive issues, physical appearance and communication (48). The PedsQL Multidimensional Fatigue Scale measures fatigue through 18 items in 3 domains: general fatigue, sleep/rest fatigue, and cognitive fatigue (48). The PedsQL questionnaires use a five-point Likert scale: 0 = never a problem; 1 = almost never a problem; 2 = sometimes a problem; 3 = often a problem; 4 = almost always a problem.

Parental HRQoL is measured by means of a Short Form Survey Instrument (RAND 36-item Health Survey) (49). It consists of 36 items divided into 8 domains (Physical functioning, limitations due to physical health, limitations due to emotional health, energy/fatigue, emotional well-being, social functioning, pain, and general health). All items are scored on a 5-point Likert scale from 0 to 100, with 100 representing the highest level of functioning possible. Aggregate scores are compiled as a percentage of the total points possible, using the RAND scoring table. The scores from the items within each domain are averaged, for a final score per domain (49). A physical and a mental component summary score can be calculated but not a total score (49).

### Additional Interview and Observations

#### Semi-structured Interview

Interviews take place with the intervention group children and parents within two weeks post intervention. The interview guide is semi-structured, with open-ended questions to help understand parents' and children's experiences and perspectives (50, 51). The interview guide is attached as Supplementary File 2. The interview guide seeks to explore the children's confidence in movement, joy of movement and social interactions. The interviewer asks the child questions such as: “What activities do you do at the hospital?”; “Can you tell me what you think is fun to play?”; “Can you tell me what you think is boring to play?”; or “Who do you play with?”. The parents are asked questions within the same focus sphere, such as: “How is your child's gross motor function right now?”; “Have you seen any changes over the last 6 months?”; “How do you feel about having participated in the intervention?”; “How do you feel challenging your child to develop his/her gross motor functioning?”, “How do you experience your child's belief in his/her own abilities?”. Follow-up questions are posed to both children and parents for deeper understanding and clarification purposes. Darcy et al. (6, 52) inspired the interview modality for the participants aged 3–5 years. Each child-parent dyad is interviewed as a unit with a clear indication when the questions are directed to the parents or to the child. Playing and toys are used to break the ice and to facilitate responses from the children. When a child is hesitant to participate, the interviewer directs questions only to the parents while the child can join in at any desired moment (6). Only the parents are interviewed when the child is younger than 3 years old, however, those children are encouraged to join in if they desire.

#### Observation

Observations are made during the active play sessions to get an understanding of how structured active play impacts the development of the children's social and personal skills and focuses on confidence in movement, joy of movement and social interactions. Observations are made over the first 12 months of the intervention when conducted at the hospital. The intervention research team members are considered participant observers during the sessions as they both actively participate while simultaneously observing the children (53). This active insider-observer perspective is selected to allow upfront observation of the children. Written notes are consequently made directly following each session and then reformulated into scenic descriptions, a narrative-inspired observation method (54, 55). Scenic descriptions create nuanced insight and understanding of what occurs during session activities. The researcher's voice can be present in the descriptions, however, he/she must remain true to what is experienced (54, 56, 57).

### Sample Size

#### RCT

No studies have been identified in the literature that investigate the potential effectiveness of a structured active play intervention on gross motor function in preschool children diagnosed with cancer. Hence, the sample size for the current study is based on a study that investigated the effectiveness of a structured active play intervention on gross motor function in healthy preschool children (21). Based on the mean and standard deviation (SD) in the intervention group = 99.31 (9.07), and a mean (SD) in the control group = 93.24 (9.02), an Alpha = 0.05 and Power = 80%, it is estimated that 70 children are required for the study. However, to account for possible attrition, 20% more participants are included. Hence, the study includes 84 children in total (42 in each group).

#### Interviews

To ensure credibility and data adequacy, a minimum sample of 20 families will be interviewed at T3 (post-intervention, primary end-point) (51, 58, 59). Broad representation will be attempted by including all age groups, diagnostic groups and family structures (50, 60, 61). However, a convenience sampling strategy will be applied. Only the intervention group families are invited to be interviewed with the project team, within two weeks post-intervention.

### Analysis

#### Statistical Considerations

The collected data will be analyzed based on intention-to-treat (ITT). The treatment effect difference will be estimated through constrained longitudinal data analysis that constrains the mean at baseline to be equal between the arms. Constrained linear mixed models will be applied in two scenarios. In the 1st scenario, predictors include follow-up time points categorized as T2 (3 months after treatment initiation) and T3 (6 months after initiation) to account for any non-linear effect and dummy variables representing the intervention group at T2 and T3 follow-ups, respectively. In the 2nd scenario, the time variable will be treated as a continuous variable and interaction between groups and the time variable will be included instead. Baseline characteristics such as age (as a continuous variable) and cancer type (hematologic malignancy, tumors located in the central nervous system (CNS tumor), and extracranial solid tumors) will be included additionally as covariates in both scenarios. Patient identity serves as a random intercept. Likelihood ratio tests based on maximal likelihood will be applied for model selection of the fixed effects to determine linear or non-linear associations. A significance level at 0.05 will be used.

#### Thematic Analysis

Interviews will be transcribed verbatim and the observation notes will be transformed into scenic descriptions. Thematic analysis will be conducted on all data (interview and scenic description transcripts) (62). The analysis will be inductive and focuses on the children's and parents' experiences with structured active play. The interview and scenic descriptions will be analyzed independently. Both sets of qualitative data will be analyzed following four flexible steps: (1) reading the material to obtain an overall understanding; (2) identifying meaningful units; (3) coding the units; and (4) summarizing the codes into key themes (62, 63). Two researchers will conduct the analysis independently and then together, and reflect and discuss results during the process to strengthen credibility and dependability of the data and, as such, the trustworthiness of the study (50, 60). Credibility will be enhanced through the use of relevant direct quotes from the data (60).

### Data Management

All data are collected in paper form and then manually entered in a secure electronic database (REDCap) by a member of the project team. As this process can produce data management errors, the data will be cross-checked independently by two members of the project team to assure their quality.

## Discussion

This two-arm parallel, superiority randomized controlled trial (RCT), with an intervention group and a control group, can potentially examine the effects of a structured active play intervention on gross motor function in preschool children diagnosed with cancer. Additionally, it can explore the children's and parents' experiences with structured active play and how that impacts their child's gross motor, social, and personal development. This protocol reflects choices made when designing this study and considers the expected challenges, strengths and dilemmas of exploring the experience of preschool children undergoing intensive cancer treatment in parallel with an intervention and securing viable outcome measurements.

Testing children is generally difficult and testing preschool children (1 to 5 years old) diagnosed with cancer is expected to be even more challenging due to their burdened physical and mental state. Testing for objective data in this age group and the results of these tests will vary on any given day in correlation with the child's level of well-being (e.g., treatment-related side effects, including pain or nausea, etc.), mood (including mood changes related to dexamethasone treatment) and ability to understand instructions and to cooperate. However, as we are aware of this methodological challenge, various efforts have been made to optimize the opportunity of obtaining data, such as: (1) having an assessor who is an experienced pediatric physiotherapist assigned to the patient group and when performing the gross motor function tests; (2) creating an environment in which the children feel a sense of familiarity and security; and (3) including parent proxy assessments. To create a trusting testing environment, parents are present during all tests as they often provide both familiarity and security for their child. We also prioritized having experienced physiotherapists familiar to the participant group as outcome assessors to increase the probability of successfully obtaining data. This outweighed having blinded outcome assessors. To further strengthen findings from this study a secondary outcome that measures gross motor function is selected (i.e., the PEDI). This is a subjective parent proxy means of reporting the child's level of everyday function.

With regards to the intervention design, the research team developed an intervention program targeting preschoolers' gross motor function through structured active play. Similar to above, conducting this type of activity intervention within the preschooler age group is challenging given developmental considerations and disease characteristics. The study intervention uses a structured active play approach. Play is fundamental for children, fun and provides an opportunity for them to create and explore a world they can master (26). When using play in physical activity it is possible to stimulate imagination and have fun using gross motor skills in a way that appeals to the child. Instead of asking a child to practice balance and build leg strength by merely jumping, a scenario can be created in which the child can imagine being a frog jumping through a forest. Designing a structured active play intervention, while allowing for individualization, also means compromising and forgoing a strict protocol. The trade-off is reduced reproducibility. When playing with children, it is necessary to be open to their imagination and be prepared to spontaneously change an activity in order to entice them. Not all children are motivated by the same thing and this raises the question of whether it is possible to describe exactly what the intervention comprises. This intervention uses three theoretical core principles, including: ritual practices, repetition and challenge; all aiming to establish recognizability, security and motivation for the children. These components also ensure that all the sessions are identically structured, with the same starting and ending rituals, repeated activities and challenges. Exact activities performed during each session are documented.

Gross motor function in preschoolers is developed through interaction with others (children, parents or other adults) and reinforced through activities. The intervention is therefore designed to allow intervention group children and parents to participate together in the in-hospital sessions, when possible. Moreover, the exercise professional conducting the sessions at the hospital as well as parents and siblings participate by either following, supporting the child or leading in an activity. Whether moving across the floor like an animal or balancing through an obstacle course, each party participates. Active participation by parents during the sessions can also reinforce the parents' ability to carry out the same activities with their child at home. Participation with others in activities is not only essential in supporting the child's physical development but also his/her social and personal skills. Group activities necessitate patience, ability to take turns and to cooperate with others.

Finally, a strength of this study is that it will collect long-term data. Being physically active is correlated with optimal gross motor function development and the latter is associated with social-, cognitive-, language- and personal- development in healthy preschool children (12–14, 27). The longitudinal relationship between gross motor function and health shows that reduced motor function competence during childhood is associated with higher body fat, lower cardiorespiratory fitness and lower physical fitness later in life (64, 65). Studies of children diagnosed with cancer, aged 1–18 years show that gross motor function impairments are still present two years after diagnosis and up to seven years post-treatment (5, 66). Research in physical activity and preschoolers diagnosed with cancer is important to better understand how physical activity can play a role in counteracting impairments of gross motor function and increase the possibility of sustaining normal everyday activities during and after treatment.

This protocol describes a study that will contribute by providing data on differences in gross motor function between preschoolers diagnosed with cancer who participate in an early initiated structured active play intervention and children diagnosed with cancer who are receiving standard care. Findings from this study, once completed, will also provide insights into the intervention group children's and parents' experiences with structured active play. If the trial proves the intervention to be effective, structured active play may become a standard rehabilitation component and findings from the study may be transferable to children with other chronic diseases and those experiencing long-term hospitalization.

## Ethics and Dissemination

The study complies with the ethical research principles described in the Declaration of Helsinki II. The study has been peer-reviewed and approved by the National Committee on Health Ethics Research through the Regional Research Ethics Committee in the Capital Region of Denmark (jr.nr.: H-20023949), and data handling is approved by the Danish Data Protection Agency (jr. nr.: P-2020-290). Written informed consent is obtained from parents prior to inclusion in the study and parents are informed that participation is voluntary and that their child or themselves can drop out of the study at any time.

### Safety and Adverse Events

Previous studies have shown physical activity is found safe for children with cancer, regardless of diagnosis (2, 4, 5, 11, 67). Nevertheless, all adverse events defined as unintended negative consequences that are experienced during the structured active play sessions are documented, despite the possibility of a causal relationship between the intervention and usual care (68).

### Dissemination Policy

Future results will be presented in peer-reviewed scientific journals and at international conferences. Authorship eligibility will follow the Vancouver Recommendations for authorships.

## Data Availability Statement

The datasets generated and/or analyzed during the current study are not publicly available due Danish and EU personal data legislation but are available from the corresponding author on reasonable request.

## Ethics Statement

The studies involving human participants were reviewed and approved by National Committee on Health Ethics Research through the Regional Research Ethics Committee in the Capital Region of Denmark (jr.nr.: H-20023949). Written informed consent to participate in this study was provided by the participants' legal guardian/next of kin.

## Author Contributions

The intervention content was developed by AP. The outcome measures were selected and evaluated by AP, MF, HL, PS-A, JC, and HW. The statistical analysis plan was prepared by HZ. KS and TF provided childhood cancer specific expertise. HL and MF led the project. AP wrote the initial draft of the manuscript. All authors contributed the final version, actively and substantially contributed to the design of the intervention study, and read and approved the final manuscript.

## Funding

This work is a part of Childhood Oncology Network Targeting Research, Organization & Life expectancy (CONTROL), supported by Danish Cancer Society (R-257-A14720) and the Danish Childhood Cancer Foundation (2019-5934). Furthermore, this study was specifically supported by individual grants from the Danish Cancer Society (R-284-A16272-20-S15 and R296-A16956) and the Danish Childhood Cancer Foundation (2019-5947; 2020-6756; 2021-7400). Funding sources played no role in the design or conduct of the study, collection of data, analysis, interpretation of results, preparation, review, approval of the manuscript or decision to submit the manuscript for publication.

## Conflict of Interest

The authors declare that the research was conducted in the absence of any commercial or financial relationships that could be construed as a potential conflict of interest.

## Publisher's Note

All claims expressed in this article are solely those of the authors and do not necessarily represent those of their affiliated organizations, or those of the publisher, the editors and the reviewers. Any product that may be evaluated in this article, or claim that may be made by its manufacturer, is not guaranteed or endorsed by the publisher.

## Abbreviations

PDMS-2, Peabody Developmental Motor Scales: Second Edition
PEDI: Pediatric Evaluation of Disability Inventory
6MWT: Six-minute walk test
2MWT: Two-minute walk test
HRQoL: Health-related quality of life
ITT: Intention-to-treat
TTI: Treatment initiation
CONTROL, Childhood Oncology Network Targeting Research: Organization &
Life expectancy.

## References

[1] Ness KK Kaste SC Zhu L Pui CH Jeha S Nathan PC. Skeletal, neuromuscular and fitness impairments among children with newly diagnosed acute lymphoblastic leukemia. Leuk Lymph. (2015) 56:1004–11. 10.3109/10428194.2014.944519 25030039 PMC4336225

[2] Thorsteinsson T Larsen HB Schmiegelow K Thing LF Krustrup P Pedersen MT. Cardiorespiratory fitness and physical function in children with cancer from diagnosis throughout treatment. BMJ Open Sport Exerc Med. (2017) 3:e000179. 10.1136/bmjsem-2016-000179 28761697 PMC5530132

[3] Vainionpää L. Clinical neurological findings of children with acute lymphoblastic leukaemia at diagnosis and during treatment. Eur J Pediatr. (1993) 152:115–9. 10.1007/BF02072486 8444217

[4] Nielsen MKF Christensen JF Frandsen TL Thorsteinsson T Andersen LB Christensen KB. Effects of a physical activity program from diagnosis on cardiorespiratory fitness in children with cancer: a national non-randomized controlled trial. BMC Med. (2020) 18:1–12. 10.1186/s12916-020-01634-6 32624004 PMC7336676

[5] Hartman A Winkel ML van Beek RD de Muinck Keizer-Schrama SMPF Kemper HCG van den Heuvel-Eibrink MM. A randomized trial investigating an exercise program to prevent reduction of bone mineral density and impairment of motor performance during treatment for childhood acute lymphoblastic leukemia. Pediatr Blood Cancer. (2009) 53:64–71. 10.1002/pbc.21942 19283791

[6] Darcy L Enskär K Björk M. Young children's experiences of living an everyday life with cancer – a three year interview study. Eur J Oncol Nurs. (2019) 39:1–9. 10.1016/j.ejon.2018.12.007 30850132

[7] Björk M Nordström B Hallström I. Needs of young children with cancer during their initial hospitalization: an observational study. J Pediatr Oncol Nurs. (2006) 23:210–9. 10.1177/1043454206289737 16766686

[8] Nielsen MKF Christensen JF Frandsen TL Thorsteinsson T Andersen LB Christensen KB. Testing physical function in children undergoing intense cancer treatment—a RESPECT feasibility study. Pediatr Blood Cancer. (2018) 65:1–9. 10.1002/pbc.27100 29741279

[9] Thorsteinsson T Schmiegelow K Thing LF Andersen LB Helms AS Ingersgaard MV. Classmates motivate childhood cancer patients to participate in physical activity during treatment: a qualitative study. Eur J Cancer Care. (2019) 28:1–10. 10.1111/ecc.13121 31215079

[10] Braam K Van der Torre P Takken T Veening MA Van Dulmen-den Broeder E Kaspers GJL. Physical exercise training interventions for children and young adults during and after treatment for childhood cancer (Review) SUMMARY OF FINDINGS FOR THE MAIN COMPARISON. Cochrane Database Syst Rev. (2016) 3:CD008796. 10.1002/14651858.CD008796.pub3 27030386 PMC6464400

[11] Fiuza-Luces C Padilla JR Soares-Miranda L Santana-Sosa E Quiroga J V., Santos-Lozano A, et al. Exercise intervention in pediatric patients with solid tumors: the physical activity in pediatric cancer trial. Med Sci Sports Exerc. (2017) 49:223–30. 10.1249/MSS.0000000000001094 27631396

[12] WOLD HEALTH ORGANIZATION,. Guidelines on Physical Activity, Sedentary Behaviour Sleep. World Health Organization (2019). p. 4. Available online at: https://apps.who.int/iris/bitstream/handle/10665/325147/WHO-NMH-PND-2019.4-eng.pdf?sequence=1&isAllowed=y%0Ahttp://www.who.int/iris/handle/10665/311664%0Ahttps://apps.who.int/iris/handle/10665/325147

[13] Timmons BW Leblanc AG Carson V Gorber SC Dillman C Janssen I. Systematic review of physical activity and health in the early years (aged 0-4 years). Appl Physiol Nutr Metab. (2012) 37:773–92. 10.1139/h2012-070 22765840

[14] Barnett LM Salmon J Hesketh KD. More active pre-school children have better motor competence at school starting age: an observational cohort study. BMC Public Health. (2016) 16:1–8. 10.1186/s12889-016-3742-1 27724941 PMC5057262

[15] Clark JE. On the problem of motor skill development. J Phys Educ Recreat Danc. (2007) 78:39–44. 10.1080/07303084.2007.10598023

[16] Logan SW Robinson LE Wilson AE Lucas WA. Getting the fundamentals of movement: a meta-analysis of the effectiveness of motor skill interventions in children. Child Care Health Dev. (2012) 38:305–15. 10.1111/j.1365-2214.2011.01307.x 21880055

[17] Williams HG Pfeiffer KA O'Neill JR Dowda M McIver KL Brown WH. Motor skill performance and physical activity in preschool children. Obesity. (2008) 16:1421–6. 10.1038/oby.2008.214 18388895

[18] Wrotniak BH Epstein LH Dorn JM Jones KE Kondilis VA. The relationship between motor proficiency and physical activity in children. Pediatrics. (2006) 118:e1758–65. 10.1542/peds.2006-0742 17142498

[19] Piek JP Baynam GB Barrett NC. The relationship between fine and gross motor ability, self-perceptions and self-worth in children and adolescents. Hum Mov Sci. (2006) 25:65–75. 10.1016/j.humov.2005.10.011 16442171

[20] Garcia C Garcia L Floyd J Lawson J. Improving public health through early childhood movement programs. J Phys Educ Recreat Danc. (2002) 73:27–31. 10.1080/07303084.2002.10605876

[21] Bellows LL Davies PL Anderson J Kennedy C. Effectiveness of a physical activity intervention for head start preschoolers: a randomized intervention study. Am J Occup Ther. (2013) 67:28–36. 10.5014/ajot.2013.005777 23245780 PMC3722665

[22] Zeng N Ayyub M Sun H Wen X Xiang P Gao Z. Effects of physical activity on motor skills and cognitive development in E.: GCU library resources - all subjects. Biomed Res Int. (2017) 2017:1–13. 10.1155/2017/2760716 29387718 PMC5745693

[23] Veldman SLC Jones RA Okely AD. Efficacy of gross motor skill interventions in young children: an updated systematic review. BMJ Open Sport Exerc Med. (2016) 2:e000067. 10.1136/bmjsem-2015-000067 27900154 PMC5117028

[24] Reilly JJ Kelly LA Montgomery C Willamson A Fisher A McColl JH. Physical activity to prevent obesity in young children: cluster randomised controlled trial. Bmj. (2006) 333:1171. 10.1136/bmj.38979.623773.55 17028105 PMC1647320

[25] Pellegrini AD Smith PK. Physical activity play: the nature and function of a neglected aspect of play. Child Dev. (1998) 69:577–98. 10.1111/j.1467-8624.1998.tb06226.x 9680672

[26] Ginsburg KR Shifrin DL Broughton DD Dreyer BP Milteer RM Mulligan DA. The importance of play in promoting healthy child development and maintaining strong parent-child bonds. Pediatrics. (2007) 119:182–91. 10.1542/peds.2006-2697 17200287

[27] Leonard HC Hill EL. Review: the impact of motor development on typical and atypical social cognition and language: a systematic review. Child Adolesc Ment Health. (2014) 19:163–70. 10.1111/camh.12055 32878369

[28] Hart S (editor). Inclusion, Play and Empathy: Neuroaffective Development in Children's Groups. 1st ed. London; Philadelphia, PA: Jessica Kingsley Publisher (2017).

[29] Bjorklund DF Brown RD. Physical play and cognitive development: integrating activity, cognition, and education. Child Dev. (1998) 69:604–6. 10.1111/j.1467-8624.1998.tb06229.x 9680673

[30] Hart S. Inclusion, play and empathy: neuroaffective development in children's groups. 1 ed. In: Hart S, editor. Jessica Kingsley Publisher (2017). Available online at: http://bcu.summon.serialssolutions.com/2.0.0/link/0/eLvHCXMwbV07T8MwED5Bu3SDAiJtqTIx0cqN4zzGpGpgQ0KF1UpiW0JqqwrRIfz63jkPoOpo2Tq_73Tn-z4DcG_OZic6wfdDLbjmqtSLwjA81blhMTNFwXnJjAXMrYK3Z3-difdfhiFy57fVZ5cShJuMexULHkb-o8WxVlhucTQ_Ws2LsmXar6sv0R1jlOr3ukjac0akeAJ

[31] Chan A Tetzlaff JM Altman DG Laupacis A Gøtzsche PC Krleza-Jeric K. SPIRIT 2013 Statement: defining standard protocol items for clinical trials | The EQUATOR Network. Ann Intern Med. (2016) 158:200–7. 10.7326/0003-4819-158-3-201302050-00583 23295957 PMC5114123

[32] Henderson K Glancy M Little S. Putting the fun into physical activity. J Phys Educ Recreat Danc. (1999) 70:43–5. 10.1080/07303084.1999.10605706

[33] Spagnola M Fiese BH. Family routines and rituals. Encycl Hum Relations. (2013) 20:284–99. 10.1097/01.IYC.0000290352.32170.5a

[34] University of Minnesota. Developmental Skills for Ages 2 to 3 Years. Fairview Heal Serv. (2010). p. 3–6. Available online at: https://www.fairview.org/fv/groups/internet/documents/web_content/developmen_201009262104505.pdf

[35] University of Minnesota. Developmental Skills for Ages 4 to 5 Years. Fairview Heal Serv. (2010). Available online at: https://www.fairview.org/fv/groups/internet/documents/web_content/developmen_2010092621054611.pdf

[36] Schmidt-Andersen P Møller T Mogensen PR Schmiegelow K Larsen HB Nielsen MKF. Feasibility and validity of the actiheart activity monitor in children who were hospitalized with cancer coadmitted with classmates: a RESPECT study. Pediatr Phys Ther. (2020) 32:226–33. 10.1097/PEP.0000000000000712 32604366

[37] Wilson RW Jacobsen PB Fields KK. Pilot study of a home-based aerobic exercise program for sedentary cancer survivors treated with hematopoietic stem cell transplantation. Bone Marrow Transplant. (2005) 35:721–7. 10.1038/sj.bmt.1704815 15696182

[38] Bowen DJ Kreuter M Spring B Linnan L Weiner D Bakken S. How we design feasibility studies. Am J Prev Med. (2009) 36:452–7. 10.1016/j.amepre.2009.02.002 19362699 PMC2859314

[39] Folio MR, Fewell, RR,. Peabody Developmental Motor Scales - Second Edition - Examiner's Manual. 2nd ed. Austin, TX: PRO-ED (2000). Available online at: https://onlinelibrary.wiley.com/doi/10.1002/9780470373699.speced1552

[40] Wang HH Liao HF Hsieh CL. Reliability, sensitivity to change, and responsiveness of the Peabody Developmental Motor Scales-Second Edition for children with cerebral palsy. Phys Ther. (2006) 86:1351–9. 10.2522/ptj.20050259 17012639

[41] Stahlhut M Christensen J Aadahl M. Applicability and intrarespondent reliability of the pediatric evaluation of disability inventory in a random danish sample. Pediatr Phys Ther. (2010) 22:161–9. 10.1097/PEP.0b013e3181dbf965 20473099

[42] Berg M Jahnsen R Frøslie KF Hussain A Berg M Frøslie KF. Reliability of the pediatric evaluation of disability inventory (PEDI). Phys Occup Ther Pediatr. (2004) 24:61–77. 10.1300/J006v24n03_05 15257969

[43] Neumann S Kwisda S Krettek C Gaulke R. Comparison of the grip strength using the martin-vigorimeter and the JAMAR-dynamometer: establishment of normal values. In Vivo. (2017) 31:917–24. 10.21873/invivo.11147 28882959 PMC5656866

[44] Robertson A Deitz J. A Description of gripstrength in preschool children. Am J Occup Ther. (1987) 42:647–52. 10.5014/ajot.42.10.647 3202109

[45] Enright PL. The six-minute walk test. Respir Care. (2003) 48:783–5. 12890299

[46] Bohannon RW Wang YC Bubela D Gershon RC. Normative two-minute walk test distances for boys and girls 3 to 17 years of age. Phys Occup Ther Pediatr. (2018) 38:39–45. 10.1080/01942638.2016.1261981 28129009

[47] Bohannon RW Bubela D Magasi S McCreath H Wang YC Reuben D. Comparison of walking performance over the first 2 minutes and the full 6 minutes of the Six-Minute Walk Test. BMC Res Notes. (2014) 7:1–6. 10.1186/1756-0500-7-269 24767634 PMC4012174

[48] Varni JW Burwinkle TM Katz ER Meeske K Dickinson P. The PedsQL^TM^ in pediatric cancer: reliability and validity of the pediatric quality of life inventory^TM^ generic core scales, multidimensional fatigue scale, and cancer module. Cancer. (2002) 94:2090–106. 10.1002/cncr.10428 11932914

[49] Patel AA Donegan D Albert T. The 36-Ltem short form. J Am Acad Orthop Surg. (2007) 15:126–34. 10.5435/00124635-200702000-00007 17277259

[50] Lincoln YS Guba EG. But is it rigorous? Trustworthiness and authenticity in naturalistic evaluation. New Dir Progr Eval. (1986) 1986:73–84. 10.1002/ev.1427

[51] Smith B Sparkes AC. Interview - qualitative interviewing in the sport and exercise sciences. In: Smith B, Sparkes AC, editors. Routledge Handbook of Qualitative Research in Sport and Exercise. 1st ed. London; New York, NY: Routledge (2017). p. 103–23.

[52] Darcy L Björk M Enskär K Knutsson S. The process of striving for an ordinary, everyday life, in young children living with cancer, at six months and one year post diagnosis. Eur J Oncol Nurs. (2014) 18:605–12. 10.1016/j.ejon.2014.06.006 24997519

[53] Ciesielska M Broström KW Ölhander M. Observation Methods. In: Ciesielska M, Jemielniak D, editors. Qualitative Methodologies in Organization Studies. Cham: Palgrave Macmillan (2018). p. 33–52.

[54] Winther H. Pratitioner research. In: Thing LF, Ottesen L, editors. Methods in Sport and Physioterapy Research. 2 ed. Copenhagen: Munksgaard. (2015). p. 172–89.

[55] Winther H. Let's move: embodiment, leadership, and dance in education. In: Tin MB, Telseth F, Tangen JO, Giulianotti R, editors. The Nordic Model and Physical Culture. Routledge Reasearch in Sport, Culture and Society. 1 ed. Oxon; New York, NY: Routhledge (2020). p. 51–67.

[56] Sparkes AC. Researching the senses in sport and exercise. In: Smith B, Sparkes A, editors. Routledge Handbook of Qualitative Research in Sport and Exercise. New York, NY: Routledge (2017). p. 343–54.

[57] Thorpe H Olive R. Conducting observations in sport and exercise settings. In: Smith B, Sparkes AC, editors. Routledge Handbook of Qualitative Research in Sport and Exercise. 1st ed. London; New York, NY: Routledge (2017). p. 124–38.

[58] Morse JM. Data were saturated. Qual Health Res. (2015) 25:587–8. 10.1177/1049732315576699 25829508

[59] Vasileiou K Barnett J Thorpe S Young T. Characterising and justifying sample size sufficiency in interview-based studies: systematic analysis of qualitative health research over a 15-year period. BMC Med Res Methodol. (2018) 18:1–18. 10.1186/s12874-018-0594-7 30463515 PMC6249736

[60] Graneheim UH Lundman B. Qualitative content analysis in nursing research: concepts, procedures and measures to achieve trustworthiness. Nurse Educ Today. (2004) 24:105–12. 10.1016/j.nedt.2003.10.001 14769454

[61] Patton M. Purposeful sampling. In: Qualitative Evaluation and Research Methods. Beverly Hills, CA: Sage (1990). p. 169–86.

[62] Braun V Clarke V. Using thematic analysis in psychology. Qual Res Psychol. (2006) 3:77–101. 10.1191/1478088706qp063oa

[63] Malterud K. Systematic text condensation: a strategy for qualitative analysis. Scand J Public Health. (2012) 40:795–805. 10.1177/1403494812465030 23221918

[64] Lima RA Bugge A Ersbøll AK Stodden DF Andersen LB. The longitudinal relationship between motor competence and measures of fatness and fitness from childhood into adolescence. J Pediatr. (2019) 95:482–8. 10.1016/j.jped.2018.02.010 29782811

[65] Utesch T Bardid F Büsch D Strauss B. The relationship between motor competence and physical fitness from early childhood to early adulthood: a meta-analysis. Sport Med. (2019) 49:541–51. 10.1007/s40279-019-01068-y 30747376

[66] Hartman A Van Bos C Den Stijnen T Pieters R. Decrease in motor performance in children with cancer is independent of the cumulative dose of vincristine. Cancer. (2006) 106:1395–401. 10.1002/cncr.21706 16453332

[67] Morales JS Santana-Sosa E Santos-Lozano A Baño-Rodrigo A Valenzuela PL Rincón-Castanedo C. Inhospital exercise benefits in childhood cancer: a prospective cohort study. Scand J Med Sci Sport. (2020) 30:126–34. 10.1111/sms.13545 31482597

[68] FDA. What is a Serious Adverse Event?. FDA (2016). Available online at: https://www.fda.gov/safety/reporting-serious-problems-fda/what-serious-adverse-event~

