# Supplementary material for: Rehabilitation Including Structured Active Play for Preschoolers With Cancer (RePlay)—Study Protocol for a Randomized Controlled Trial
Source: Front Pediatr. 2022 May 9;10:834512. doi: 10.3389/fped.2022.834512 (PMC9124960; doi:10.3389/fped.2022.834512)
Supplement: Supplementary file 1 [file Data_Sheet_1.PDF]

Introduction

Inspirational guidance for motivating your child during active treatment

This structured 'Active play' kit contains inspirational material for play and movement activities that can be done at home or in a hospital room.
Physical activity is vital for your child's gross motor development as well as to enhance social and personal skills.

The kit contains:

1) **Play and movement activity descriptions**
2) **Songs, including accompanying movement actions**
3) **Basic movement skills description cards**
4) **Animal bingo board game, including cards**

The kit also contains **a logbook** for activity registration.

All activities are **color-coded**:

● **Orange** ● **Purple** ● **Blue**

The colors represent different stages of gross motor development for age groups 1-2 years old and 3-5 years old. It may be necessary to focus on **orange** and **purple** activities prior to moving to the **blue** activities, depending on how quickly your child's skill level advances — this is a natural occurrence.

It is important that younger children remain as physically active as possible during the day and that activities vary - so you are encouraged to use the material as inspiration for that purpose.

Everyday activities are also ideal to encourage movement. Your child could:

- walk (at home or when hospitalized, use corridors);
- visit the playground;
- climb stairs; and
- pick up toys or tidy the room after playtime.

The material allows your child to select activities that appeal to him/her.

Color-coded gross motor development stages

1-2 years old

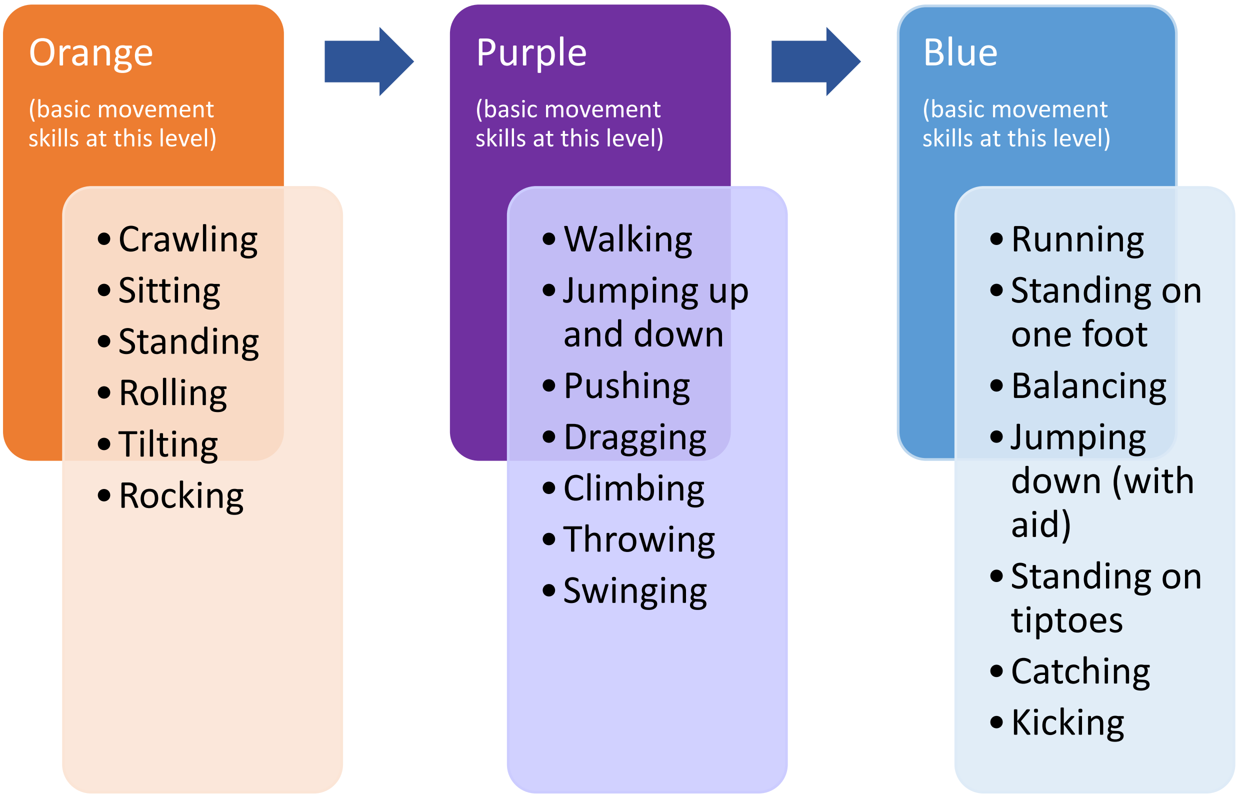

References: University of Minnesota. Developmental Skills for Ages 2 to 3 Years. Fairview Heal Serv [Internet]. 2010;3–6. Available from: https://www.fairview.org/fv/groups/internet/documents/web_content/developmen_201009262104505.pdf; University of Minnesota. Developmental Skills for Ages 4 to 5 Years. Fairview Heal Serv [Internet]. 2010; Available from: https://www.fairview.org/fv/groups/internet/documents/web_content/developmen_2010092621054611.pdf.

Color-coded gross motor development stages

3-5 years old

Orange

(basic movement skills at this level)

- Crawling
- Sitting
- Standing
- Rolling
- Tilting
- Rocking
- Swinging
- Walking
- Jumping up and down
- Pushing
- Dragging
- Climbing
- Throwing
- Running
- Standing on tiptoes

Purple

(basic movement skills at this level)

- Jumping down
- Standing on one foot
- Walking on tiptoes
- Kicking
- Catching
- Balancing
- Running

Blue

(basic movement skills at this level)

- Hopping on one foot
- Standing on one foot for an extended period
- Skipping
- Swinging alone
- Kicking with force

References: University of Minnesota. Developmental Skills for Ages 2 to 3 Years. Fairview Heal Serv [Internet]. 2010;3–6. Available from: https://www.fairview.org/fv/groups/internet/documents/web_content/developmen_201009262104505.pdf; University of Minnesota. Developmental Skills for Ages 4 to 5 Years. Fairview Heal Serv [Internet]. 2010; Available from: https://www.fairview.org/fv/groups/internet/documents/web_content/developmen_2010092621054611.pdf.

Examples of different activities

1-2 years old

Rolling a ball

Sit across from each other, with legs apart. Roll a ball back and forth from one to the other using big and small balls.

Hide and seek using toys

Hide toys around the room in a way that best encourages your child to crawl and move maximally. Use various toys.

Mountain climber

Use the furniture (sofa, coffee table, stools, pillows) to climb and jump on.

Play with a balloon

Sit or stand across from each other and throw the balloon back and forth without it touching the floor.

Obstacle course

Make an obstacle course with whatever is available (stools, sofa, blankets, pillows, etc.). The obstacle course can include, e.g. crawling under the table or up onto the sofa or balancing on a pillow – your imagination is the only limit.

Running

Run indoors or outdoors. Running can take the form of a race or use the animal cards to try to run as fast as the animals depicted.

Examples of different activities

3-5 years old

Move like an animal

Using the animal cards, take turns at selecting one. Move across the room like the animal that your card depicts, e.g. stand on tiptoes to depict a tall giraffe; leap like a frog; wriggle like a snake.

Play with a balloon

Sit or stand across from each other and throw the balloon back and forth without it touching the floor.

Obstacle course

Make an obstacle course with whatever is available (stools, sofa, blankets, pillows, etc.). The obstacle course can include, e.g. crawling under the table or up onto the sofa or balancing on a pillow – your imagination is the only limit.

Dancing

Put on your favorite music and dance together.

Tag Games

Play different kinds of tag games.

Races and relays

Race against each other – e.g. "who can come first to the kitchen", "… to the tree", "…to the other side of the room".
Do relay races - e.g., each player stands at opposite ends of the room, with three toys. See who is faster at moving all three toys, one at a time, to the opposite end of the room.
