# Supplementary material for: Rehabilitation Including Structured Active Play for Preschoolers With Cancer (RePlay)—Study Protocol for a Randomized Controlled Trial
Source: Front Pediatr. 2022 May 9;10:834512. doi: 10.3389/fped.2022.834512 (PMC9124960; doi:10.3389/fped.2022.834512)
Supplement: Supplementary file 2 [file Data_Sheet_2.PDF]

Interview guide for semi-structured interviews for the RePlay Project

<u>Interview with the child:</u>

Subject	Question	Extra opening question
**Playing at home**	**Do you like to play?**<br>- What **do you like** to play? Why?<br><br>- What **do you not like** to play? Why?<br><br>**What do you play at home?**<br>Who do you play it with?<br><br>**Do you sometimes play outside?**<br>- What do you play outside?<br>- Who do you play it with?	Do you have your own room at home?<br>- What do you play in your room?
**Playing at the hospital**	**Do you play when you are at the hospital?**<br>- **What** do you play?<br>- **Who** do you play it with?<br><br>- **Have you ever played with me?**<br>- Do you remember what we played?<br>- Was it fun or not?<br><br>**When do you not feel like playing?**	Is it sometimes hard to play when you are at the hospital?

<u>Interview with the parents</u>

**Subject**	**Questions**	**Elaborative questions**
**Intro questions**	Please introduce us to your family	
**The child's gross motor and physical function**	**What do you think is the current status of your child's gross motor and physical functioning?**<br><br>Can you describe what has changed over the last six months regarding your child's gross motor and physical functioning?<br><br>How do you perceive your child's desire to be active?<br><br>Describe how your child has been active while at home?	- Can you elaborate?<br>- In what way?<br><br>Is there anything in particular that you have noticed?<br><br>How do you experience your child's belief in his/her own body – does he/she believe that his/her body has the strength and ability to do what he/she wants?<br><br>Does your child have a good sense of his/her own physical abilities?<br><br>If your child faces a challenge when moving or doing an activity, how does he/she react?
**The intervention – the physical and social elements**	**Can you share your experience with the structured active play project at the hospital?**<br><br>**Do you believe that the intervention impacted your child?**<br>- In what way?	- Did you feel it was important to you as a family to participate?<br>• Why/why not?<br><br>What are your observations of your child's participation in the project?<br><br>Do you think the intervention positively impacted your child's gross motor functions?<br>- In what way?

	**What has it meant for you as a family to have participated in the intervention?**<br><br>**Did you use the inspirational guidance material?**<br>- How did you use it?	Did the intervention challenge your child's gross motor functional level?<br>- In what way<br><br>How do you think your child responded to those challenges?<br><br>How did you feel about the presence of an instructor?
**Future considerations**	**What are your expectations in relation to future activities for your child?**<br><br>**Moving forward, do you feel confident with motivating your child to continue being physical active?**	
